# Implementation of morbidity and mortality conference in a community hospital NICU and narrative review

**DOI:** 10.3389/fped.2023.1321296

**Published:** 2023-12-01

**Authors:** Christina Chan, Christine Pazandak, Dimitrios Angelis

**Affiliations:** Division of Neonatal-Perinatal Medicine, Southwestern Medical Center, University of Texas Dallas, TX, United States

**Keywords:** neonatology, case review, morbidity and mortality review, quality improvement, community hospital

## Abstract

**Background:**

The process of morbidity and mortality review (MMR) is recognized as an essential component of quality improvement, patient safety, attitudes towards patient safety, and continuing education. Despite the common use of MMR for all disciplines of medical care, recommendations have not been published regarding the implementation of MMR in a community hospital setting in the United States.

**Objectives:**

Review the literature on MMR conferences. Describe the implementation of an MMR conference in a community hospital neonatal intensive care unit (NICU).

**Conclusions:**

The establishment of a case overview method of MMR is feasible for a community hospital NICU. It increases staff and physician group awareness and education over common and complex mortality and morbidity etiologies, improves staff participation with unit management, links case presentation with open discussion and action items, and identifies opportunities for systemic changes to improve patient care.

## Introduction

The practice of medicine is advanced by self-reflection and a desire to improve care of patients through refinement of procedures and medical regimens. The first systematic record keeping tool used for improving care was utilized in the late 1800s according to archived records of Cushing and Codman (1). Since that time, the medical record has evolved to include granular information on every patient encounter. This information can be used to find controllable etiologies for morbidity and mortality.

**Figure 1:**
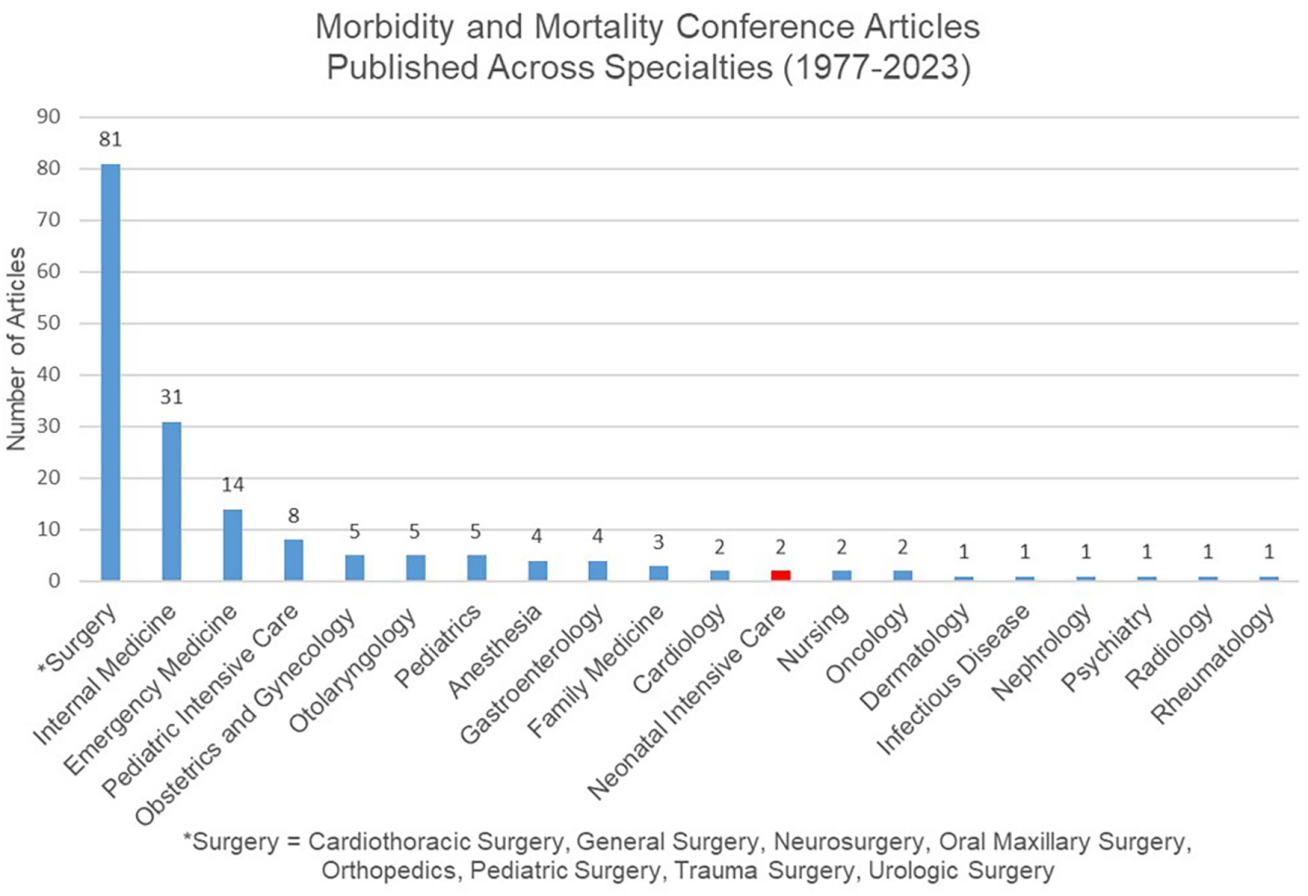
Distribution of number of articles discussing morbidity and mortality conferences across medical and surgical specialties (1977–2023).

Despite the history of morbidity and mortality review (MMR), there is limited information published on guidelines for community hospitals in the United States, and there are no published recommendations on how to format an MMR conference for a community hospital NICU. An advanced search of PubMed on 6/8/2023 using the search terms “morbidity and mortality conference” located in the title and/or abstract with filters for English language and human species found 175 published articles. As demonstrated in Figure 1, the surgical specialties most commonly referenced “morbidity and mortality conference” in their publications (81 articles) followed by internal medicine (31 articles) and emergency medicine (14 articles). A few articles were written from an interdisciplinary perspective including surgery and internal medicine disciplines (2) while other articles provided general recommendations for all medical disciplines (3–8). Although there has been increasing recognition in adult literature of the need to improve the morbidity and mortality conference process, limited attention has been given to this topic in pediatric literature.

Of the articles published in the pediatric literature, only a few addressed neonatal medicine and these articles focused on utilizing the morbidity and mortality conference as a vehicle for education of end-of-life issues and follow-up of neonatal deaths on transports (9–11). Of those, only one discussed recommendations on how to conduct and improve morbidity and mortality conferences, but this study was not conducted within a community hospital NICU (11).

### MMR conference nuances and challenges

The Institute of Medicine created a report, “To Err is Human: Building a Safer Health System,” which described the need for healthcare organizations to evaluate their current practice in order to provide safe care (12). While this is an ongoing conversation, intrinsic barriers to the process include: (1) Time, (2) Lack of full representation from all service lines, (3) Fear of blame, and (4) Lack of institutional support.

### MMR in community practice settings

In community practice settings, the MMR conference is often managed by hospital-based performance evaluation or unit-based quality committees reviewing reported (often by nursing or other auxiliary staff reporting on physician behavior) or triggered events (mortalities, significant morbidities, or hospital determined metrics). The primary goal is to identify opportunities to avoid harm/error and improve the quality of patient care. Attendance at these meetings is often restricted to physicians with recommendations distributed to various stakeholders following the meeting.

#### MMR in teaching facilities

The primary goals regarding the teaching facility MMR are three pronged: (a) identify and avoid future errors, (b) education for residents or fellows, usually giving emphasis to interesting cases rather than systemic issues, (c) identify opportunities for culture improvement in a unit or a department. Of note, MMRs are required by the Joint Commission and for this reason have been implemented in the normal operational routine of teaching facilities as part of the Accreditation Council of Graduate Medical Education (ACGME). MMR conferences should be linked with quality improvement initiatives, which is more difficult in teaching facilities with a primary focus on “interesting cases,” educational or rare cases, rather than cases where meaningful systemic changes in practice can be found (13).

MMR conferences have been routinely used by academic surgical programs to improve trainee performance. The overarching goals for surgical MMR conferences include education and identification of quality improvement opportunities, but the format may vary widely based on practice location (9, 14–17). Academic intensive care groups frequently adopt an in-depth case review (IDR) model that is owned and managed by trainees and focuses on investigating 1–2 cases with similarities for opportunities to improve process or care (14, 18, 19). In academic institutions, most attendees for MMR conferences are trainees (medical students, residents, fellows) or physician faculty (14). Physician assistants or nurse practitioners may also attend. Nursing staff or respiratory therapists are typically less involved in MMR.

Use of survey tools to assess participant engagement and evaluation of the MMR process has been shown to result in significant improvement in surgical and internal medicine MMR conferences at academic facilities (20, 21).

#### MMR and department structure

Logistically larger departments need standardization of case selection for presentation and may incorporate randomization in selection. In addition, routine feedback needs to be given to the quality improvement committee of these departments so systemic process issues can be followed up and the improvement process can be monitored. Smaller departments can follow the teaching model that ACGME requires, as more time may be available for each case (13). While there are historic differences between the methods of case presentation between surgical and internal medicine MMR conferences, there is increasing convergence toward identification of systemic issues contributing to poor patient outcomes. Surgical conferences classically focus on individual error, while medical conferences focus on systemic contributions over individual error (22). This focus on individual culpability in surgical conferences is shifting as surgical programs participate in national quality initiatives such as the National Surgical Quality Improvement Program (NSQIP). In addition to offering comparisons in outcomes using risk adjustment models, the NSQIP has also urged surgical programs to abandon the MMR conference structure (23, 24).

#### Methods to identify hidden morbidity

Efforts to close the gap between graduate medical education and hospital quality projects are implemented in some instances by linking resident-initiated conferences addressing safety issues to quality initiatives (25). For example, resident presentations of “minor” complications have value in the education of residents on the effect of system gaps on patient outcomes (26). Other authors in surgical specialties suggest using the “unexpected outcome approach”, which has been proposed as a reasonable way to identify errors after extensive data analysis (27). In these cases, statistical tools (Youden's Index, J-statistics) assess if the unwanted outcome can be explained by its baseline incidence. In other words, these tools help define reasonable standardized cut-off points or optimal thresholds for side effects (28). The use of fish-bone diagrams, group assessments of unwanted outcomes, and root cause analyses are other tools that may help to uncover known and hidden patient morbidities. These tools are time intensive and may be prohibitive when evaluating large case numbers.

Peer review processes and resistance to change: The peer review process is mandatory after a medical complication. Unfortunately, this is not always feasible in academic MMR conferences. The peer review requires representation from those directly involved with the event in question so that accurate information can be obtained and a plan for change can be developed. In MMR conferences at teaching facilities, there can be an overemphasis on the discussion with educational generalizations while conclusions and opportunities for improvement may be overlooked.

Resistance to change is one of the most difficult issues facing the MMR. Nussenbaum et al. (16) suggests four interventions to encourage culture change: extensive root analysis, application of Reason's Swiss Cheese models, utilization of a Just Culture (providers are accountable for their own actions only), and the substitution test (question if same action would be taken by the evaluator) (29). Some authors suggest independent observers and members of quality committees be involved in these conferences. They also recommend that members of the team who resist change attend MMR conferences in different departments (13). Many of these techniques require protected time and additional resources to execute in a timely manner and may not be feasible in community hospital settings with limited support.

## Implementation

### A new MMR conference: positive change in a level 3 NICU

This is a description of how we developed a pragmatic, evolving, and increasingly comprehensive MMR (ecMMR) conference for use in a community hospital NICU. Our clinical setting is a community hospital with a wide variety of community obstetric practices who monitor and deliver infants of women with high-risk pregnancies while the neonatal site is staffed by faculty of a single academic practice without the regular involvement of trainees. The pediatric services at this hospital are limited to normal newborn care and neonatal intensive care.

From 2015 to 2018, a traditional academic IDR conference format was used. This discussion was limited to 1–2 cases per month that were presented by a physician who was not directly involved with the cases. This method only allowed for a maximum of 24 cases to be discussed each year. The physician presenting the cases would select the cases with some informal feedback from the medical director. The audience was primarily made up of physicians with intermittent nursing leadership presence. The average attendance was 5–6 people. Feedback of the cases were derived from the physician reviewer, with rare audience amendments. Participation from nursing, respiratory therapy, and auxiliary staff was minimal. No continuing medical education (CME) credits were available.

On review of this process, a decision was made to change from IDR to an ecMMR presentation. Prior to initiation of the new ecMMR presentation in 2019, major morbidities and mortalities were found to be occurring in the unit. The process was owned by the medical director who solicited and reviewed the electronic medical records using Epic queries, approached on-service physicians, and encouraged nursing leaders to note of any cases involving preidentified morbidities and mortalities. The preidentified morbidities and mortalities were based on metrics tracked by the Vermont Oxford Network. This ecMMR was set at the same time monthly, with an in-person and virtual option provided. All disciplines practicing in the NICU were invited and welcomed to participate. Group participation was elicited by a series of simple questions and attendees collectively determined the final recommendation from the case. A member of the hospital quality and risk committee and nursing supervisory leadership were also invited to attend. This linked the ecMMR conference with the hospital chain of quality and review. Yearly reviews of the process were then completed, and metrics were adjusted annually to identify opportunities for improvement. This process was identified as an ethics education meeting and CME credits for physicians, nursing, and respiratory therapy were made available.

On review of the process in 2022, the ecMMR conference method increased case review to 311 cases for that year with an average of 26 cases per session. Attendance diversity increased to include surgical attendings, pharmacy, nutrition, bedside nursing staff, and bedside respiratory therapists with comments and questions addressed in real time. Attendance numbers also increased to 186 over the course of the year with an average of 15 participants per session.

## Recommendations and suggestions

**Figure 2:**
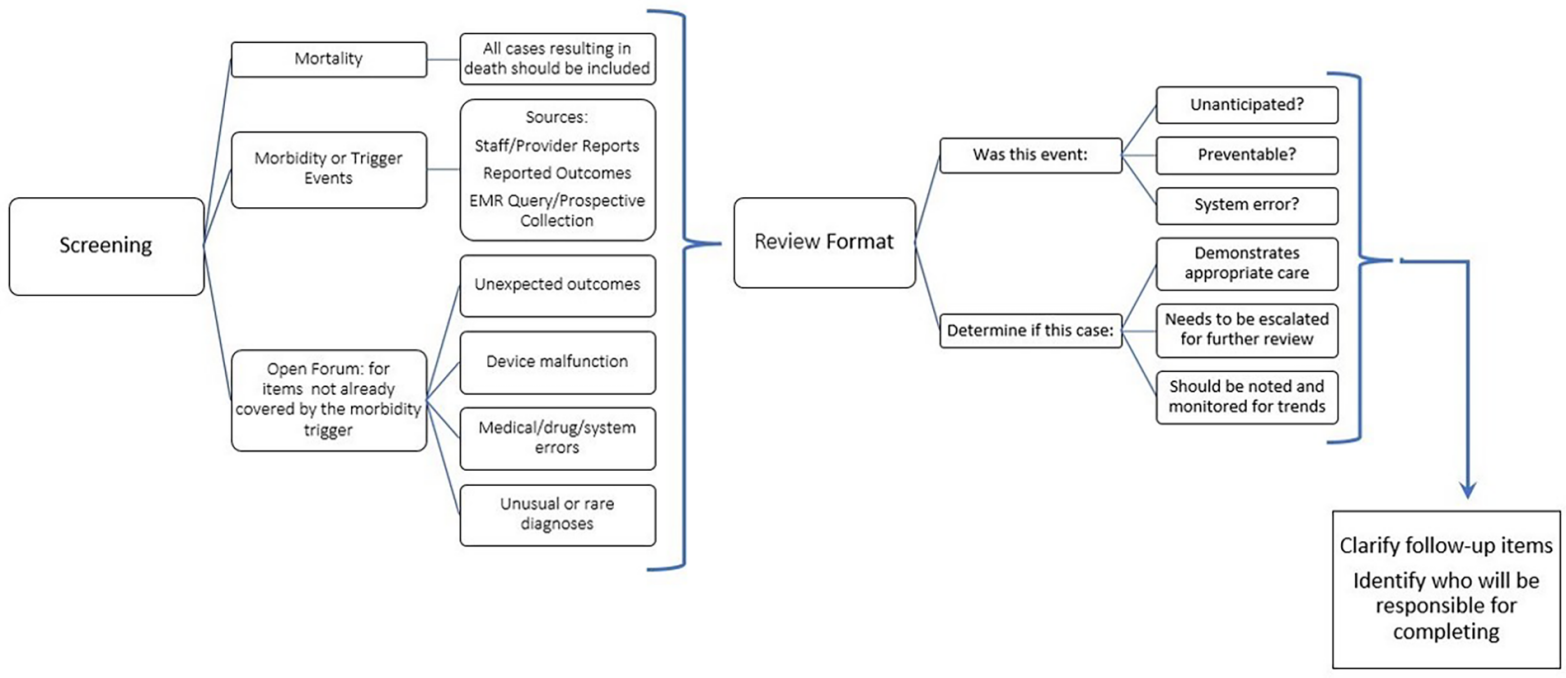
Flow chart showing overview of case presentation conference for community hospital NICU.

We present 3 basic principles for the development of an evolving, comprehensive and successful MMR. These pragmatic principles could be used by a community-based neonatal program as clinical guidelines to establish an ecMMR. We describe the overview of our case presentation in Figure 2.

1. Designate a point person or team.
2. Case selection and presentation structure designed to comprehensively evaluate cases, detect patterns, and uncover both obvious root causes but also hidden opportunities for individual and systemic improvements.
3. Create an environment of safety.

### Designation of team

A mature person with diverse clinical experience and a specific interest in improving patient care is ideal for this role. This individual should be supported with consistent protected time to allow for thoughtful case preparation for each meeting. A team of people may be helpful depending on the size of the unit. As our program has grown, we have designated (1) a data extraction person who identifies the cases and outlines the clinical information, (2) a meeting organizer who coordinates the meetings, takes attendance, takes notes, and arranges for CME, (3) a case presenter who reviews the case and leads the discussion, and (4) a manager who closes the loop and follows up on practices changes that are suggested during the conference.

Case presentation by an objective team member is ideal but can be burdensome in a community hospital setting where the time required for preparation is not financially supported. In our community hospital setting, presentation by the medical director was found to be expedient. To create a safe space for open discussion, we started each meeting with a statement of purpose: describing the protected nature of these discussions and emphasizing that this is a team building activity designed to promote unified approaches, identify areas of improvement, and provide better patient care.

### Case selection and presentation

Comprehensive case selection is often difficult, especially in community hospital settings. When the primary job of most team members is providing direct clinical care, this limits time for data extraction.

Depending on the unit resources, the selection methods may need to be integrated over time or pragmatically selected to be successful:

1. Mortality cases are often widely known, easy to identify, and should be discussed as a foundational component for an ecMMR. If the mortality case has preventable components, it also often identifies the highest yield opportunities for improvement. This is limited in scope and occurs infrequently in community hospital settings.
2. Staff or provider reported cases are often how cases are selected. This addresses immediate unit concerns and builds unit consciousness about specific cases. However, this method is often limited in scope, will not capture subtle issues or trends, and are often biased to rare or unusual case presentations.
3. Morbidity metrics are commonly recorded for NICU patients especially for those units participating in national databases including the Pediatrix BabySteps Data Warehouse, Vermont Oxford Network, and the Children's Hospital Neonatal Consortium. These lists can provide comprehensive evaluation of neonatal outcomes frequently uncovering hidden opportunities for improvement. However, these reports are often delayed and removed from staff and provider experiences thus missing opportunities to address staff and providers’ immediate concerns.
4. Electronic medical record (EMR) query or dashboards from hospital records may be an ideal way to identify all potential morbidity. However, this relies on diagnostic descriptions and diagnostic tags which may not be added or appropriately indicated in the hospital chart which can limit this method of case identification.

#### Presentation of cases

The discussions of each case were focused on system-based malfunctions, avoiding if possible conflict or blame amongst providers. Because all cases were opened for group discussion, for cases where the medical director was the physician caring for the patient, other physicians and staff were actively encouraged to provide feedback. Cases where evaluation was deemed not objective could be referred to the hospital practitioner performance evaluation committee where physicians and practitioners from other disciplines review and adjudicate cases through the formal hospital reporting system. Items for follow-up were identified and a designee to follow-up was identified in real time. Incidents where exceptional care was provided were identified as well and noted during conversation and emphasized as opportunities for group learning on best practice. Annually, these cases are reviewed, and metrics presented to the unit. In open conversation, opportunities for system enhancement, unit education, and equipment needs are discussed and presented to unit and hospital leadership. Detailed cumulative statistics for trends of major outcomes also provide an opportunity to retrospectively define adverse effects (near miss, medical error, harm etc.) and use of root cause analyses tools such as the Ishikawa, fishbone analysis tool were reserved for cases where systemic issues were suspected (ex. Recurrent CLABSI events) (30).

### Create an environment of safety

As previously mentioned, our case presentations start with a statement of purpose to remind the team and educate any new members that the purpose of these quality improvement protected conversations is to provide the team a way to understand practice and improve our team approach to patient care. Through a focus on system etiologies and solutions and on patient outcomes, conversations are kept professional. While individual education may be indicated, it was often appropriate to address prior to the case presentation with mention that individual education had been performed prior to the discussion during the ecMMR. Once systems-based issues and individual responsibilities are delineated, clear documentation of actions after a complication was completed. If individual responsibilities were identified, then in-depth peer review would follow.

As the operation of the NICU changed over time, outcomes identified for improvement also changed. Periodically, additional morbidities were identified and added to the tracking sheet to follow over time; other morbidities became rarer and were subsequently dropped from the tracking sheet to allow for a more robust discussion of other findings. The cases collected and how they are identified, presented, and addressed have shifted in a plan-do-study-act cycle with multidisciplinary guidance from the team.

## Conclusion

In this report, we are the first to describe a paradigm of a successful change in the M&M conference process in a community-based hospital neonatal intensive care unit in the United States. We believe that in this ecMMR model which includes a more comprehensive discussion of cases rather than IDR, transparency is improved since all potential cases of interest can be included in the discussion and all service lines in the unit are welcome to participate. We have found that this method triggers QI/QA initiatives in many areas. An obvious risk of limiting the time per case is the decreased educational time, and hence less time to review the literature and transfer knowledge to the providers. We address this by organizing educational sessions not linked to the case review.

The establishment of an ecMMR for neonatal outcomes is feasible for a community hospital neonatal intensive care unit. This approach increases awareness and education over common and complex mortality and morbidity. It improves staff participation with unit management by linking case presentation with open discussion and action items, and identifying systemic issues. Further evaluation should be done to evaluate the benefits to patient outcomes in the community practice neonatal intensive care setting.

## Data Availability

The original contributions presented in the study are included in the article/Supplementary Material, further inquiries can be directed to the corresponding author.
